# Quantifying the Spatiotemporal Heterogeneity of PM_2.5_ Pollution and Its Determinants in 273 Cities in China

**DOI:** 10.3390/ijerph20021183

**Published:** 2023-01-09

**Authors:** Li Yang, Chunyan Qin, Ke Li, Chuxiong Deng, Yaojun Liu

**Affiliations:** 1 College of Tourism, Hunan Normal University, Changsha 410081, China; 2 College of Geographic Sciences, Hunan Normal University, Changsha 410081, China; 3 College of Mathematics & Statistics, Hunan Normal University, Changsha 410081, China

**Keywords:** PM_2.5_, spatiotemporal heterogeneity, variation characteristics, determinants, GTWR, China

## Abstract

Fine particulate matter (PM_2.5_) pollution brings great negative impacts to human health and social development. From the perspective of heterogeneity and the combination of national and urban analysis, this study aims to investigate the variation patterns of PM_2.5_ pollution and its determinants, using geographically and temporally weighted regression (GTWR) in 273 Chinese cities from 2015 to 2019. A comprehensive analytical framework was established, composed of 14 determinants from multi-dimensions, including population, economic development, technology, and natural conditions. The results indicated that: (1) PM_2.5_ pollution was most severe in winter and the least severe in summer, while the monthly, daily, and hourly variations showed “U”-shaped, pulse-shaped and “W”-shaped patterns; (2) Coastal cities in southeast China have better air quality than other cities, and the interaction between determinants enhanced the spatial disequilibrium of PM_2.5_ pollution; (3) The determinants showed significant heterogeneity on PM_2.5_ pollution—specifically, population density, trade openness, the secondary industry, and invention patents exhibited the strongest positive impacts on PM_2.5_ pollution in the North China Plain. Relative humidity, precipitation and per capita GDP were more effective in improving atmospheric quality in cities with serious PM_2.5_ pollution. Altitude and the proportion of built-up areas showed strong effects in western China. These findings will be conductive to formulating targeted and differentiated prevention strategies for regional air pollution control.

## 1. Introduction

China has undergone persistent and serious atmospheric pollution in the past [1]. In 2015, 265 of 338 (78.4%) prefecture-level cities exceeded the national ambient air quality standards. In particular, PM_2.5_ (a complex particle with a diameter of ≤2.5 μm) is the primary atmospheric particulate pollutant [2], which can endanger human health and interfere with social development [3,4,5,6]. In 2016, the mortality related to exposure to PM_2.5_ represented about 9.98% of the total reported deaths in China [7]. In 2017, the cost of health hazard induced by PM_2.5_ exposure was 3344.8 billion Yuan, accounting for 3.85% of GDP in China [8]. Therefore, exploring the characteristics and determinants of PM_2.5_ concentrations will be of great significance to formulate effective prevention and control strategies.

Previous studies have long been concerned with the variation characteristics of PM_2.5_ concentrations and examined different scales. For instance, Yang and Christakos [9] evaluated PM_2.5_ variations from the dimension of time and space in the Shandong province in 2014. Shen et al. [10] detected the distribution characteristics of PM_2.5_ pollution in Chinese representative urban agglomerations from 2015 to 2017. Zhou et al. [11] studied the evolution characteristics of atmospheric pollution in 337 cities in China between 2015 and 2019. Luo et al. [12] discussed the variations of PM_2.5_ concentrations in Harbin in 2017, revealing that air quality exhibited a significant “weekend effect” in time.

Numerous studies have worked to explore the determinants of PM_2.5_ concentrations. For instance, Li et al. [13] established the automatic computer algorithms to explore the meteorological formation conditions conducive to PM_2.5_ concentrations in north China. Cai et al. [14] concluded that the frequent haze in Beijing was probably attributed to the circulation changes induced by the greenhouse gas discharge. Some scholars discussed that the weather conditions exhibited a stronger influence when PM_2.5_ pollution was at a higher level [15]. In evaluating the effect of anthropogenic determinants on urban PM_2.5_ levels in China, Jiang et al. [16] demonstrated that industrial structures exhibited the strongest driving force. Zhou et al. [17] revealed that wind direction determined the spatial patterns of PM_2.5_ pollution, and that the spillover effects of anthropogenic variables were more significant in the North China Plain. Yang et al. [18] compared the strength of natural and socioeconomic environments on PM_2.5_ concentrations, revealing that the natural environment performed a stronger role in China.

Various methods have been employed to quantify the driving forces of PM2.5 concentrations. There were two main types of methods, one of which was global regression models adopted to analyze the average influence of determinants in a single region with prominent air pollution or the nationwide, such as the dynamic panel model [19], the spatial econometric method [20], and the structural equation model [21]. As a country with unbalanced regional development and prominent geographical diversity, different indicators and their links with PM_2.5_ pollution may exhibit differences across China. Therefore, the second was local regression models applied to detect the influences of different indicators on PM_2.5_ concentrations in different regions or cities, such as geographically weighted regression (GWR) [22], geographically and temporally weighted regression (GTWR) [23,24]. Wang et al. [25] adopted the global regression models and GWR to explore the variations and driving forces of PM_2.5_ pollution in 2014, realizing the combination of global regression and local regression.

To sum up, previous studies have fully examined the determinants of PM_2.5_ concentrations and have achieved rich results. However, the mechanism of atmospheric pollution is complicated [26]. Both the global regression models and the local regression models can only analyze the force of a single determinant, and exclude the consideration of the interaction between determinants. At present, the interaction analysis of determinants of PM_2.5_ pollution is limited to the study of the nation as a whole [27] or the regions with prominent air pollution [28]. Few studies have analyzed the interaction and spatiotemporal heterogeneity of determinants of air pollution from national and local perspectives. In this sense, this paper combines the advantages of the geographical detector technique and the GTWR model to enrich the current literature. A comprehensive analytical framework was established, composed of 14 determinants from multiple dimensions. To be specific, this paper investigated the mechanism of the spatial disequilibrium of PM_2.5_ concentrations in China between 2015 and 2019 from a national perspective. The interaction among determinants of spatial disequilibrium of PM_2.5_ concentrations was explored. Secondly, this study evaluated the strength of different determinants on PM_2.5_ pollution in various cities, using the GTWR model from a local regression perspective. Additionally, with the help of the analysis framework of the IPAT (Human Impact Population Affluence Technology) model [29], the current paper selected nine socioeconomic indicators that affect PM_2.5_ pollution from three dimensions, including population, economic development, and technology; five key natural factors were combined in the framework. The use of this framework was conducive to examining the possible causes of PM_2.5_ pollution. 

The innovativeness of this study mainly lies in the following aspects. Firstly, we considered the spatiotemporal heterogeneity of the influencing factors and realized the combination of global and local analysis, finding that variables exhibited weak temporal heterogeneity and obvious spatial heterogeneity on PM_2.5_ pollution. Furthermore, this study revealed that there were heterogeneous impacts of driving forces on the local and global levels. Additionally, interactions between the influencing factors were explored, revealing that there were complex interactions and coupling relationships between different factors on the spatial disequilibrium of PM_2.5_ pollution, with an effect of “1 + 1 > 2”. The objective of this paper is to detect the spatiotemporal heterogeneity and influencing factors of PM_2.5_ concentrations from the national and urban levels, thus providing inspiration for developing targeted and differentiated prevention strategies for current air pollution control.

## 2. Materials and Methods

PM_2.5_ data were available from China National Environmental Monitoring Center (http://www.cnemc.cn, accessed on 29 June 2021). According to the Ambient Air Quality Standard (GB3095-2012) of China for the validity of PM_2.5_ data, we excluded records with missing or invalid PM_2.5_ h values in the original data [30]. Considering that the number of monitoring stations varies in different cities, this study took the average observations of the stations located in the city to represent a city’s PM_2.5_ pollution. Through the above processing, the PM_2.5_ monitoring sites finally selected in 2015, 2016, 2017, 2018, and 2019 were distributed in 363, 361, 364, 362, and 360 cities across China. Referring to prior studies [31,32], the Kriging interpolation was adopted to reflect the spatial patterns of the yearly mean of PM_2.5_ concentrations in China between 2015 and 2019.

Ehrlich and Holdren [33] put forward the classic environmental pressure equation, namely I = PAT, which describes the effects of the population (P), economic development (A), and technology (T) on environmental (I) factors [34]. Referring to the IPAT analysis framework and according to previous findings [35,36,37,38,39,40], combined with the availability of socioeconomic data at the urban level, this paper selected nine socioeconomic indicators that affected PM_2.5_ pollution from three dimensions, including population, economic development, and technology. Specially, this paper used population density (PD, described here in terms of the ratio of total resident population to total area) to represent the population factor; per capita GDP (PCGDP), the degree of trade openness (TO), urbanization rate (UR), the proportion of built-up areas (BU), greening rate of built-up area (BUG), and industrial structure (IS) to represent economic development; and electricity consumption (EC) and the number index of invention patents granted (IP) to represent technology.

Among the nine socioeconomic factors (Table 1), the index of the number of invention patents granted was obtained from the Center for Enterprise Research of Peking University (https://opendata.pku.edu.cn/dataverse/pkucer, accessed on 29 June 2021). Other socioeconomic data were available from the 2016–2020 China City Statistical Yearbook and the corresponding China Province Statistical Yearbook. Finally, the socioeconomic data of 273 cities were obtained to match PM_2.5_ data and natural data. The units and abbreviations of each indicator are shown in Table 1.

Natural conditions play an important role in atmospheric quality by affecting the emissions, chemical reaction rate, and transport of air pollutants [41]. Based on the meteorology theory and previous studies [42,43,44,45,46], this study considered five natural factors—namely, altitude (DEM), precipitation (PRE), wind speed (WS), relative humidity (RH), and temperature (TEM) (Table 1). DEM data came from the CGIAR Consortium for Spatial Information website (http://srtm.csi.cgiar.org, accessed on 29 June 2021); PRE, WS, RH, TEM data were obtained from the China Meteorological Administration (http://data.cma.cn/, accessed on 29 June 2021; the basic data came from 699 meteorological monitoring stations, captured from 2015 to 2019. The Inverse Distance Weighted interpolation was applied to form annual meteorological data for 273 cities.

As an important method to investigate the mechanism of the spatial distribution of geographic elements, the geographical detector technique is widely adopted to explore spatial differentiation and uncover its potential determinants [47]. The factor detector is adopted to test whether a specific element is the cause of the differentiation of PM_2.5_ concentrations in space. This model is presented as follows [48]:

(1) $$q=1-\frac{1}{N\sigma^{2}}\sum_{h=1}^{L}N_{h}\sigma_{h}^{2}$$

In the formula, *q* as the explanation intensity of the differentiation of PM_2.5_ ranges from 0 to 1. The degree of differentiation for PM_2.5_ depends on the value of *q* and changes in the same direction. The model requires the independent variables to be categorized, rather than numerical; the natural breaks classification method was adopted to classify each variable into *L* layers; and the categorized independent variables were matched with PM_2.5_ data. *h* is the integer between 1 and L. *N* and $N_{h}$ represent the quantity of cities in the whole area or the layer *h*, and $\sigma^{2}$ and $\sigma_{h}^{2}\;$ are the variances.

The interaction detector can explore the interactions between various factors. The specific calculation process is to initially obtain the *q* value of two variables, namely *q*(*X1*) and *q*(*X2*) from equation (1). Hence, overlaying the two factor layers generated a new layer, namely *X1*∩*X2* and ∩, which stands for the intersection between the two factor layers [49]. Then, *q* (*X1*∩*X2*) can be obtained from equation (1). Based on the links among *q* (*X1*), *q* (*X2*) and *q* (*X1*∩*X2*), the types of interactions are “weaken, univariate” (the interaction between the two variables is less than the minimum or within the maximum and minimum), “enhanced, bivariate” (the interaction between the two variables is greater than the maximum),”independent” (the interaction between the two variables equals to the sum of them), and ”nonlinearly enhance” (the interaction is greater than the sum of the two variables).

Differing from the traditional ordinary least squares (OLS) method, which neglects the spatial correlation and spatial heterogeneity that may exist in the data, Fotheringham et al. [50] first put forward the GWR model. By introducing spatial weights, local regression of the study area can be realized through the GWR. To further consider the comprehensive effects of spatiotemporal factors, Huang et al. [51] extended the GTWR to combine time coordinate to conduct spatiotemporal heterogeneity of variables. The specific formula of the GTWR model is presented as below:

(2) $$y_{i}=\beta_{0}(u_{i},v_{i},t_{i})+\sum \beta_{k}(u_{i},v_{i},t_{i})x_{ki}+\xi_{i}$$

where $y_{i}$ denotes the value of PM_2.5_ in city *i*; ($u_{i},v_{i},t_{i}$) represents the spatiotemporal coordinate; $\beta_{0}(u_{i},v_{i},t_{i})$ denotes the intercept value; $\;x_{ki}$ denotes the k at city *i*; $\xi_{i}$ is the error term; $\;\beta_{k}(u_{i},v_{i},t_{i})\;$ is the coefficient of variable k, which is estimated as below:

(3) $$\hat{\beta }(u_{i},v_{i},t_{i})={\left[X^{T}W(u_{i},v_{i},t_{i})X\right]}^{-1}X^{T}W(u_{i},v_{i},t_{i})Y$$

where $W(u_{i},v_{i},t_{i})$ denotes the spatiotemporal weighted diagonal matrix. This study chooses the Gaussian distance decay-based function, namely:

(4) $$Wij=\exp[-\frac{{\left(d_{ij}^{ST}\right)}^{{}_{2}}}{h_{ST}^{2}}]$$

(5) $$d_{ij}^{ST}=\sqrt{\lambda [{\left(u_{i}-u_{j}\right)}^{2}+{\left(v_{i}-v_{j}\right)}^{2}]+\mu {\left(t_{i}-t_{j}\right)}^{2}}$$

where $d_{ij}^{ST}$ is the space-time distance; μ and λ as the scale indicators can reflect the influence of various spatiotemporal distances [52]; $h_{ST}$ is the bandwidth, the cross validation (CV) method is adopted to select bandwidth. The expression is:

(6) $$CV=\sum_{i}^{n}{\left[Y_{i}-{\hat{Y}}_{i}(h)\right]}^{2}$$

Among the 14 variables, to eliminate heteroscedasticity and the effects of unit changes, logarithms of non-ratio and non-index variables are taken to calculate the GTWR model. Before the regression analysis, the 14 variables were tested for multicollinearity. The variance inflation factor (VIF) of each variable in the collinearity results was less than 3, and the tolerance of each variable was less than 1, indicating that the variables selected in this study were without existing multicollinearity (Table 2).

The final model form is:

(7) $$PM_{2.5}=\beta_{0}(u_{i},v_{i},t_{i})+\sum_{j=1}^{14}\beta_{j}^{\prime }(u_{i},v_{i},t_{i})X+\xi_{i}$$

(8) $$X={\left(\ln DEM,\ln PRE,\ln WS,RH,\ln TEM,\ln PD,\ln PCGDP,UR,BU,BUG,\ln TO,IS,\ln EC,IP\right)}^{\prime }$$

ln is the logarithmic operator.

## 3. Results and Discussion

### 3.1. Temporal Changes of PM_2.5_ Concentrations

**Figure 1:**
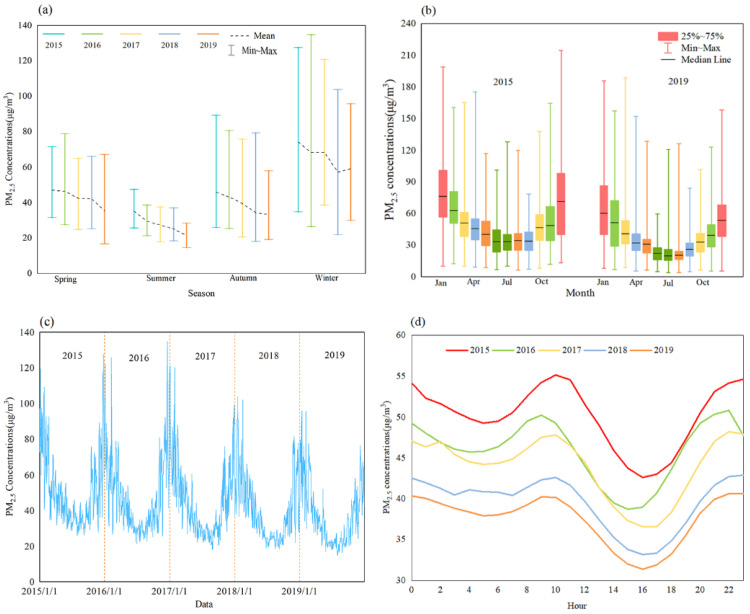
Temporal changes of PM_2.5_ in China between 2015 and 2019. (**a**): Seasonal change; (**b**): Monthly change; (**c**): Daily change; (**d**): Hourly change.

PM_2.5_ concentrations in Chinese cities decreased from 50 μg/m^3^ in 2015 to 37 μg/m^3^ in 2019. At the seasonal level, PM_2.5_ pollution was most severe in winter (January, February, and December) and least severe in summer (June to August) (Figure 1a). From 2015 to 2019, the average PM_2.5_ concentrations over four seasons fluctuated slightly, but the overall trends continued to decline, decreasing 12 μg/m^3^ in spring, 14 μg/m^3^ in summer, 13 μg/m^3^ in autumn and 15 μg/m^3^ in winter, which decreased by 24%, 38%, 7% and 20%, respectively. The monthly average variation of PM_2.5_ pollution showed a “U”-shaped pattern within a year (Figure 1b). December and January were the most polluted months, while July and August were the least polluted months. Compared to 2015, PM_2.5_ pollution in each month in 2019 has decreased significantly. Among these, December has the largest decrease, of 20 μg/m^3^, while March has the smallest decrease, of 9 μg/m^3^. PM_2.5_ concentrations in all months decreased by more than 16%. 

The daily average curve of PM_2.5_ pollution presented a pulse- type fluctuation (Figure 1c). The fluctuation cycles in winter and spring were short and frequent (about 6–8 days), but were longer and less frequent in summer and autumn (about 10–15 days). From 2015 to 2019, the daily average maximum value dropped from 135 μg/m^3^ to 90 μg/m^3^, which decreased by 33%. The daily average minimum value dropped from 26 μg/m^3^ to 12 μg/m^3^, which decreased by 53%. It shows that the air quality in Chinese cities improves gradually, while the daily average maximum value is still severe, indicating that the air quality needs to be further improved. The hourly variation showed a “W”-shaped fluctuation from 0:00 to 23:00, and decreased year by year (Figure 1d). During the day, PM_2.5_ pollution reached its peak around 10:00 and 23:00–00:00; around 06:30 and 17:00, it dropped; around 17:00, there were minimum values of PM_2.5_.

Overall, PM_2.5_ concentrations in China have dropped significantly in recent years. This decrease is linked to the implementation of the corresponding atmospheric governance strategies in the last few years [53]. For instance, the Chinese government implemented the Air Pollution Prevention and Control Action Plan 2013–2017 [54]. In 2018, the Chinese government issued the Blue Sky Protection Campaign, to reduce haze and improve air quality [55].

### 3.2. Spatial Patterns of PM_2.5_ Concentrations

**Figure 2:**
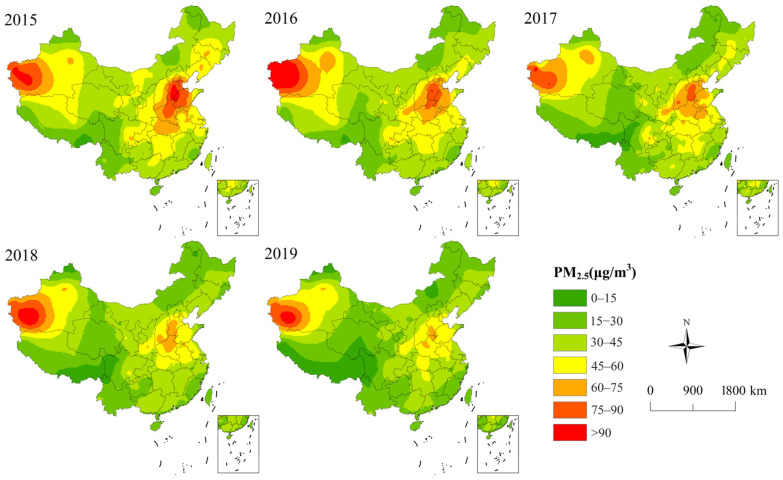
Spatial changes of PM_2.5_ in China between 2015 and 2019.

The spatial patterns of PM_2.5_ concentrations in China are shown in Figure 2. From 2015 to 2019, the overall trend of PM_2.5_ pollution in different cities was declining. Specifically, the ratio of cities lower than 15 μg/m^3^ increased from 0.55% in 2015 to 3.89% in 2019, while the ratio of cities higher than 90 μg/m^3^ decreased from 3.31% in 2015 to 0.28% in 2019. During this period, the number of cities exceeding the second level concentration limit of the environmental quality standard (35 μg/m^3^) was 288, 268, 249, 209, and 175, respectively. The most polluted cities were predominantly located in Xinjiang, the North China Plain, the Northeast Plain, and the Middle-Lower Yangtze Plain. The top 30 cities with the most serious pollution during the study period were all distributed in the north of China. Generally, PM_2.5_ pollution showed obvious north–south differentiation. The air quality in the southwest cities and southeast coastal cities was better.

### 3.3. Driving Forces of Spatial Disequilibrium of PM_2.5_ Concentrations

**Figure 3:**
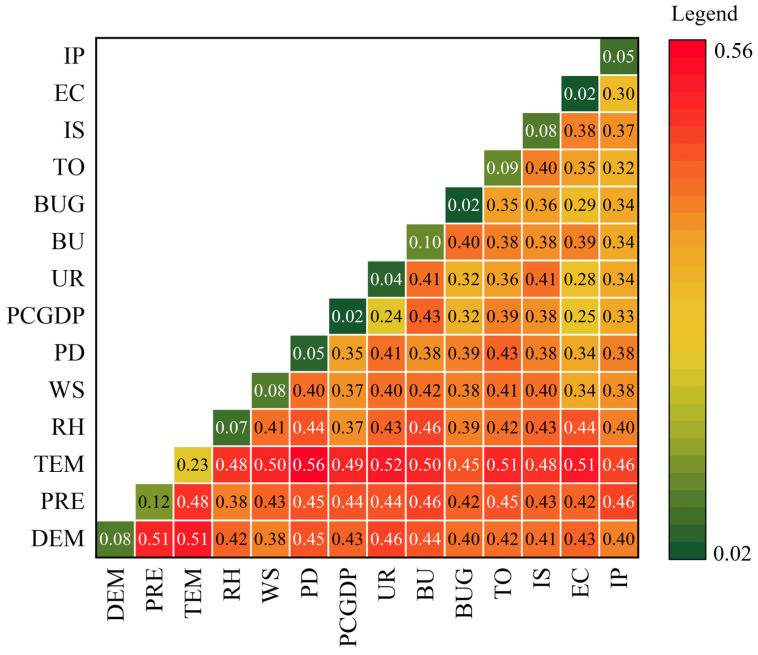
The results of geographical detector technique. Note: The main diagonal is the result of factor detector; others are interaction detector. All coefficients passed the significance test of 0.05. DEM: altitude; PRE: precipitation; TEM: temperature; RH: relative humidity; WS: wind speed; PD: population density; PCGDP: per capita GDP; UR: urbanization rate; BU: built-up area divided by urban area; BUG: greening area divided by built-up area; TO: trade openness; IS: the ratio of the added value of secondary industry; EC: electricity consumption; IP: invention patents.

From 2015 to 2019, PM_2.5_ pollution showed obvious north–south differentiation; the air quality in the southwest cities and southeast coastal cities was better. To reveal the potential mechanism of the spatial disequilibrium of PM_2.5_ pollution, this paper selected 14 variables in 273 cities from 2015 to 2019, to analyze the force of indicators and their nexus on PM_2.5_ concentrations using geographical detector technique from a national perspective (Figure 3).

From the factor detector, the top four factors that have the greatest impact on the spatial differentiation of PM_2.5_ concentrations were: temperature; precipitation; the proportion of built-up areas; and the degree of trade openness. Previous studies noted that temperature was the strongest and most stable factor affecting PM_2.5_ pollution [56,57]. According to the results of the interaction detector, all variables have the strongest interaction with temperature. The interactions between different variables were all determined to “nonlinearly enhance”—namely, the interaction of any two variables was greater than their sum, establishing an effect of “1 + 1 > 2”. This further indicated that there were complex interactions and coupling relationships between different factors on the spatial disequilibrium of PM_2.5_ concentrations, demonstrating that the effect of multiple factors should be comprehensively considered for urban PM_2.5_ control.

### 3.4. Influencing Factors of PM_2.5_ Pollution

#### 3.4.1. GTWR Regression Analysis

Since the results of geographical detector technique only provided the explanatory force of variables and their interactions on the spatial disequilibrium of PM_2.5_ pollution from a national perspective, it failed to reveal the direction of each influencing factor in different cities. Therefore, the GTWR was adopted to detect the influencing factors of PM_2.5_ concentrations in cities, serving as a supplement to the analysis results of the geographical detector technique.

**Figure 4:**
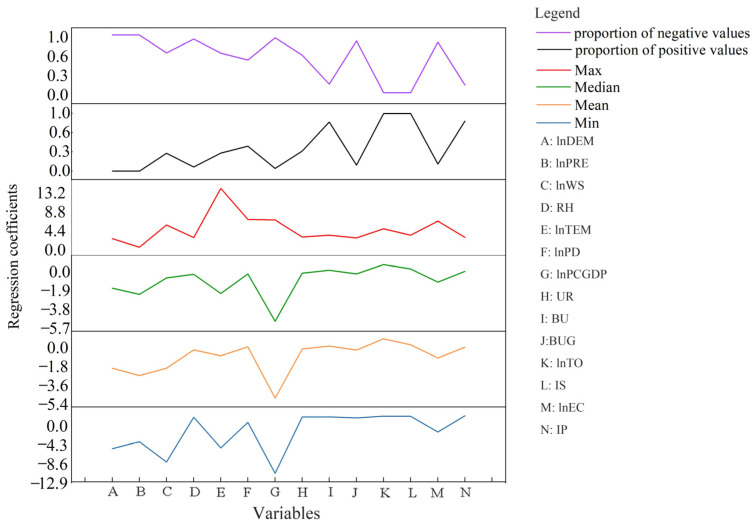
Description statistics of GTWR parameter estimation. Note: DEM: altitude; PRE: precipitation; TEM: temperature; RH: relative humidity; WS: wind speed; PD: population density; PCGDP: per capita GDP; UR: urbanization rate; BU: built-up area divided by urban area; BUG: greening area divided by built-up area; TO: trade openness; IS: the ratio of the added value of secondary industry; EC: electricity consumption; IP: invention patents.

By sorting out the regression coefficients from 2015 to 2019, six statistics are presented in Figure 4. In terms of the positive and negative ratio of the coefficients, the coefficients of altitude and precipitation in all cities were negative during the studied period; other variables had different positive and negative influences on PM_2.5_ concentrations. Influencing factors in the urban analysis showed different strengths from the national analysis, indicating that the determinants were spatially unstable and heterogeneous.

#### 3.4.2. Spatial Distribution of Regression Coefficients

In terms of time, the regression coefficients of each variable changed relatively steadily from 2015 to 2019, with weak temporal heterogeneity and obvious spatial heterogeneity. During the study period, no noticeable climate abnormalities occurred, and the socioeconomic situation was stable; thus, variables did not show significant volatility in the time dimension. Referring to previous literature of Zhang et al. [58], the average regression coefficients of each variable in each city from 2015 to 2019 are spatially visualized.

**Figure 5:**
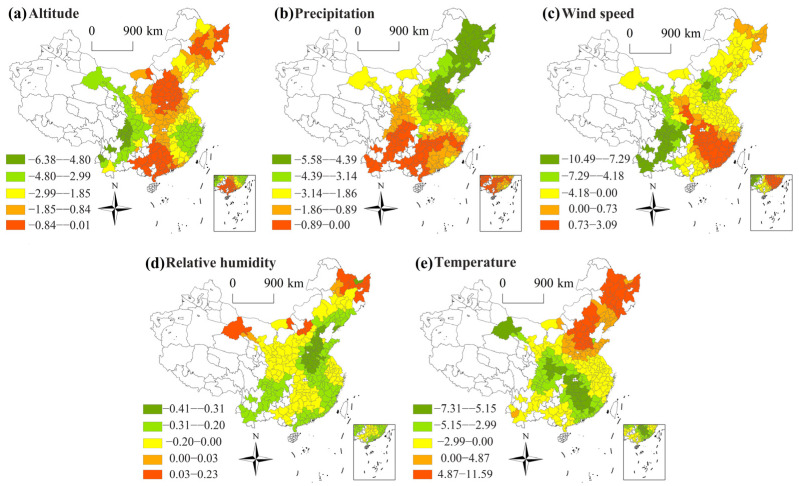
Spatial distribution of natural factors coefficients.

Figure 5 shows the spatial patterns of coefficients for natural indicators across cities. The altitude and precipitation exhibited negative links with PM_2.5_ in all cities, indicating that altitude and precipitation helped to reduce air pollution. The negative effects of altitude in central cities were smaller than those in western and eastern cities (Figure 5a). PRE in the North China Plain and the Northeast Plain exhibited the greatest negative impact on PM_2.5_ pollution, while, in southern coastal cities, altitude showed the least effect (Figure 5b). Precipitation was an important factor to effectively reduce atmospheric pollution through the washing effect [59,60]. Combined with the spatial distribution of PM_2.5_ pollution, precipitation was more effective to improve atmospheric quality in cities with serious PM_2.5_ pollution and less rainfall.

Figure 5c presents that wind speed can improve air quality in 71.06% of the cities. There was a boundary for different correlations between air pollutants and WS [61]. The moderate increase in WS promoted the diffusion of atmospheric pollution, which helped keep pollutants at lower concentrations [62], while high winds under dry climate conditions resulted in the higher probability of dust events in late winter and early spring [63], which performed a considerable role in formatting haze pollution. The positive effects were found in central China and the southeast coastal areas. Since the northwest monsoon prevails in winter in China, the severe atmospheric pollution generated in northern regions during winter would be further transported by wind direction, thus aggravating air pollution in central China and the southeast coastal areas. For the northeast region, when the wind speed reached a certain level, dust on the ground would be swept up, and the smog pollution of the atmosphere would be aggravated.

Figure 5d indicates that relative humidity can reduce PM_2.5_ concentrations in most cities (94.14%), especially in cities with more prominent pollution. According to meteorological theory, RH increased to promote efficient precipitation, which presented a scavenging impact on air pollutants [64], while the increase in relative humidity in some cities in northeast and northwest China has aggravated air pollution. This was because higher humidity promoted the hygroscopic growth of PM_2.5_ [65]. In addition, under high solar radiation and temperature, the increase in RH would accelerate secondary formation of PM_2.5_ [66,67].

Temperature exhibited a negative influence on PM_2.5_ pollution in 69.96% of cities, showing obvious north–south differences in China (Figure 5e). The impact direction of temperature on atmospheric pollutants exhibited disequilibrium under different mechanisms. The negative effect mainly lay in temperature-related air convections and the vaporization loss of PM_2.5_ [43]. Low temperatures reduced air convection and promoted the aggregation of PM_2.5_ [68]. High temperatures promoted the evaporation loss of PM_2.5_ [69], which helped to reduce PM_2.5_ pollution. While the rise of temperature lead to the increase in pollutant chemical reaction rate, which accelerated the formation of secondary PM_2.5_ [70]. Combined with the distribution characteristic of coefficients, the influences of temperature on PM_2.5_ in cities in the northeast area and Bohai region were primarily to promote the formation of secondary PM_2.5_, while the influences of temperature in other cities on PM_2.5_ were primarily to promote the evaporation loss of pollutants.

**Figure 6:**
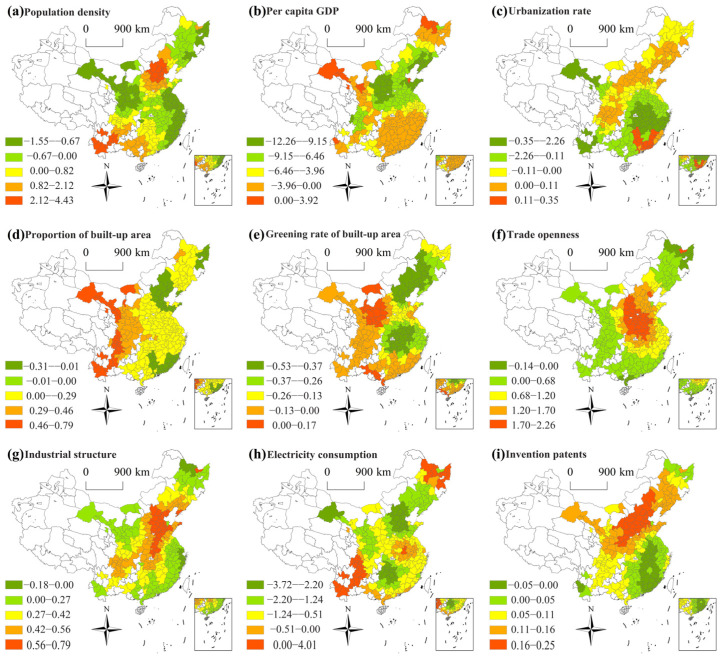
Spatial distribution of socioeconomic factors coefficients.

Figure 6 presents the spatial patterns of coefficients for socioeconomic indicators across cities. Figure 6a shows that the increase in population density has exacerbated PM_2.5_ pollution in 41.76% of cities. Higher positive coefficients were located in the BTH region and Yunnan province. For the BTH region, the increase in population density led to the increase of urban traffic flow and production scale, thereby aggravating haze pollution. Due to special geomorphology, fragile ecological environment and weak population carrying capacity, the increase in production activities attributed to the increase in population would deteriorate the ecological environment and cause atmospheric pollution in Yunnan. However, for other cities, population aggregation with an intensive and higher energy utilization could reduce PM_2.5_ pollution [71]. In addition, the increase in PD could reduce the average cost of energy consumption and public transport services, which could promote economies of density, thus reducing atmospheric pollution [72,73].

PCGDP mainly negatively influenced PM_2.5_ in studied cities (96.34%), indicating that the increase in PCGDP of these cities was not the cause of haze (Figure 6b). The potential reason for this was that the higher PCGDP promoted greater environmental awareness, which was conducive to improving air quality [74]. Combined with the distribution feature of PM_2.5_ pollution, areas with prominent PM_2.5_ pollution tended to have the greatest negative effect of PCGDP; this was because areas with severe air pollution would be subject to various restrictions, such as emission reductions, in the process of economic development, and special concern would be given to the coordination of economic growth and environmental protection. On the contrary, for the southeast coastal areas where atmospheric pollution was weak, the negative impact elasticity of economic development on atmospheric pollution was weak.

Figure 6c demonstrates that the increase in urbanization rate has reduced PM_2.5_ pollution in 68.13% of cities (Figure 6c). This finding was distinct from most current studies [75,76]. However, none of the previous studies were based on the GTWR model at the urban scale. Moreover, Luo et al. [77] found that higher urbanization helps to reduce PM_2.5_ pollution in northwest China and northeast China. In addition, Guan et al. [78] noted that rural household energy consumption was an important cause of primary PM_2.5_ pollution, and urbanization would greatly decrease this emissions component. Therefore, this finding was supported by the mechanism of PM_2.5_ discharge. The positive effects were found in the southeast coastal cities, the Bohai region, and Cheng-Yu region (Figure 6c). The potential reason lied in the relatively high level of urbanization in these areas, while the continued increase in urban population would bring about “big city diseases”, such as traffic congestion, resource shortages, and environmental pollution [79,80].

BU in most cities (82.05%) was positively correlated with PM_2.5_. The impact showed gradual weakening from the western to the eastern cities (Figure 6d). Differing from the BTH region and eastern coastal areas, the development space of the construction industry in the central and western regions was relatively large, which would cause a large amount of building dust to enter the atmospheric environment and aggravate air pollution.

Figure 6e reflects that BUG was conductive to reducing PM_2.5_ pollution in most cities (90.48%), suggesting the greening rate exhibited a mitigation impact on PM_2.5_ pollution [81]. Figure 6f presents that TO aggravated air pollution in 95.6% of cities, indicating the current trade openness has aggravated air pollution, thereby confirming the Pollution Haven Hypothesis [82,83]. Higher coefficients were distributed in north China, central China, and east China, indicating that, while developing foreign trade to attract foreign investment, China should continue to raise market access thresholds and truly implement environmental regulations.

The effect of industrial structures on PM_2.5_ pollution was positive in 96.34% of cities (Figure 6g), indicating that secondary industry showed a negative impact on air quality. Higher coefficients were located in the Cheng-Yu region and north China. The IS of the Cheng-Yu region was dominated by machinery and chemical industry, while there were plenty of energy and heavy industries in north China, which directly caused higher pollutant emissions. The coefficients in the southeast coastal area were lower, which was related to the local industrial structure, which is dominated by the tertiary industry. Figure 6h shows that electricity consumption mainly negatively affected PM_2.5_ pollution in most cities (89.01%). Compared with coal, electricity with lower emissions was an effective way to solve the dilemma of energy supply and atmospheric pollution [84]. In China, electricity was produced from fossil and non-fossil fuels. PM_2.5_ pollution would be controlled through clean retrofit of thermal power plants and gas treatment facilities [85]. The negative effects of electricity consumption in the BTH region were the most significant, the reason mainly being the replacement of residential coal consumption with natural gas and electricity for space heating in winter, to control atmospheric pollution. For instance, nearly 2.53 million households have realized the switch from coal burning stoves to natural gas or electricity stoves in rural areas around the BTH region since 2017 [84].

It was noteworthy that the innovation patents have aggravated air pollution in most cities (Figure 6i). Cities with positive high values were located in the BTH region and north China, revealing that the current invention patents of these cities failed to highlight the role of energy-saving and emission reductions. Yan et al. [86] noted that progress in technology helped to alleviate pollution, while Wang et al. [87] found that the increase in research and development spending aggravated air pollution in 72.13% of studied Chinese cities. This paper revealed that the current technological innovation in most cities was more based on the pursuit of production efficiency, rather than the impact of environmental pollution, which might cause increased consumption attributed to the rebound effect, thus deteriorating the environment [88]. Green innovation could promote the introduction and development of clean technologies, which would ensure that the entire process from production to end products has minimal environmental damage [89]. However, the adoption of green technology innovation brought obstacles to companies [90], which limited the green technology innovation behavior of the small- and medium-sized enterprises in cities [91]. When creating innovation patents, cities should emphasize the cleaner production and energy-saving and emission reductions, continuously improve patent protection mechanisms, increase incentives for green technological innovation and diffusion, and truly realize a win-win mode of technological progress and environmental protection.

Since natural factors are difficult to control, efforts to reduce air pollution should focus on socioeconomic factors. Based on its findings, this paper provides the following inspiration for developing targeted and differentiated prevention strategies for urban air pollution control. Cities in the southeast coastal areas and the BTH region should rationally guide population flow, control population size, and improve the quality of urbanization. Cities in the central region, western region and northeastern region should rationally promote the expansion of urban built-up areas to avoid air pollution caused by urban sprawl. In addition, cities in the Yangtze River Delta and the Pearl River Delta should dedicate themselves to the introduction and diffusion of green technology innovation and actively perform a demonstration effect.

Nevertheless, natural and socioeconomic environments are only part of the factors affecting PM_2.5_ pollution; other conditions, including regional pollution transport and transformation, should be further identified in future studies. In addition, more natural and socioeconomic data need to be collected to verify and enrich the current findings.

## 4. Conclusions

This paper is devoted to examining the spatiotemporal heterogeneity of PM_2.5_ pollution and its determinants from national and urban perspectives in China between 2015 and 2019. Referring to the IPAT equation to select socioeconomic factors and considering the key natural factors, this study revealed:

1. PM_2.5_ concentrations in Chinese cities declined from 50 μg/m^3^ in 2015 to 37 μg/m^3^ in 2019, exhibiting obvious regularity at different time scales. In space, PM_2.5_ pollution showed significant north–south differentiation. The air quality in the southwest cities and southeast coastal cities was better.
2. At the national level, temperature showed the greatest impact on the spatial disequilibrium of PM_2.5_ concentrations in China. The interactions between determinants enhanced the pattern, while, at the urban level, natural and socioeconomic factors exhibited weak temporal heterogeneity and significant spatial heterogeneity on PM_2.5_ pollution in different cities. Generally, population density, trade openness, secondary industry, and invention patents exhibited the strongest positive impacts on PM_2.5_ concentrations in the North China Plain. Relative humidity, precipitation and per capita GDP were more effective in improving atmospheric quality in cities with serious PM_2.5_ pollution. Altitude and the proportion of built-up areas showed strong effects in western China. Wind speed, temperature, urbanization rate, greening rate of built-up areas and electricity consumption mainly negatively affected PM_2.5_ pollution in most cities.

## Figures and Tables

**Table 1:** Definitions of variables.

Variable Set	Variables	Definition	Units
Natural factors	DEM	Altitude	m
	PRE	Total annual precipitation	mm
	WS	Yearly mean of wind speed	m/s
	RH	Yearly mean of relative humidity	%
	TEM	Yearly mean of temperature	°C
Socioeconomic factors	PD	The number of population per unit area	Person per km^2^
	PCGDP	GDP divided by total population	Yuan
	UR	Urban population divided by total population	%
	BU	Built-up area divided by urban area	%
	BUG	Greening area divided by built-up area	%
	TO	The actual use of foreign capital	Ten thousand U.S. dollars
	IS	The ratio of the added value of secondary industry	%
	EC	The electricity consumption	10,000 kWh
	IP	The number index of invention patents granted	

**Table 2:** Multicollinearity test of variables.

Variable Set	Variables	VIF	Allowance
Natural factors	lnDEM	1.481	0.675
	lnPRE	1.584	0.631
	lnWS	2.186	0.457
	RH	1.600	0.625
	lnTEM	2.440	0.410
Socioeconomic factors	lnPD	2.446	0.409
	lnPCGDP	2.734	0.366
	UR	1.324	0.755
	BU	1.283	0.779
	BUG	2.290	0.437
	lnTO	1.365	0.733
	IS	1.720	0.581
	lnEC	2.734	0.366
	IP	1.360	0.735

## Data Availability

Not applicable.

## References

[1] Li R., Wang Z., Cui L., Fu H., Zhang L., Kong L., Chen W., Chen J. (2019). Air pollution characteristics in China during 2015–2016: Spatiotemporal variations and key meteorological factors. Sci. Total Environ..

[2] Dong L., Sun W., Li F., Shi M., Meng X., Wang C., Meng M., Tang W., Liu H., Wang L. (2019). The harmful effects of acute PM2.5 exposure to the heart and a novel preventive and therapeutic function of CEOs. Sci. Rep..

[3] Lv B., Cai J., Xu B., Bai Y. (2017). Understanding the Rising Phase of the PM2.5 Concentration Evolution in Large China Cities. Sci. Rep..

[4] Yuan M., Huang Y., Shen H., Li T. (2018). Effects of urban form on haze pollution in China: Spatial regression analysis based on PM2.5 remote sensing data. Appl. Geogr..

[5] West S.E., Buker P., Ashmore M., Njoroge G., Welden N., Muhoza C., Osano P., Makau J., Njoroge P., Apondo W. (2020). Particulate matter pollution in an informal settlement in Nairobi: Using citizen science to make the invisible visible. Appl. Geogr..

[6] Verbeek T., Hincks S. (2022). The ‘just’ management of urban air pollution? A geospatial analysis of low emission zones in Brussels and London. Appl. Geogr..

[7] Maji K.J., Ye W.-F., Arora M., Nagendra S.M.S. (2018). PM2.5-related health and economic loss assessment for 338 Chinese cities. Environ. Int..

[8] Guan Y., Kang L., Wang Y., Zhang N., Ju M. (2019). Health loss attributed to PM2.5 pollution in China’s cities: Economic impact, annual change and reduction potential. J. Clean. Prod..

[9] Yang Y., Christakos G. (2015). Spatiotemporal Characterization of Ambient PM2.5 Concentrations in Shandong Province (China). Environ. Sci. Technol..

[10] Shen Y., Zhang L., Fang X., Ji H., Li X., Zhao Z. (2019). Spatiotemporal patterns of recent PM2.5 concentrations over typical urban agglomerations in China. Sci. Total Environ..

[11] Zhou D., Lin Z., Liu L., Qi J. (2021). Spatial-temporal characteristics of urban air pollution in 337 Chinese cities and their influencing factors. Environ. Sci. Pollut. Res..

[12] Luo Y., Liu S., Che L., Yu Y. (2021). Analysis of temporal spatial distribution characteristics of PM2.5 pollution and the influential meteorological factors using Big Data in Harbin, China. J. Air Waste Manag. Assoc..

[13] Li M., Wang L., Liu J., Gao W., Song T., Sun Y., Li L., Li X., Wang Y., Liu L. (2020). Exploring the regional pollution characteristics and meteorological formation mechanism of PM2.5 in North China during 2013–2017. Environ. Int..

[14] Cai W., Li K., Liao H., Wang H., Wu L. (2017). Weather conditions conducive to Beijing severe haze more frequent under climate change. Nat. Clim. Chang..

[15] Chen Z., Cai J., Gao B., Xu B., Dai S., He B., Xie X. (2017). Detecting the causality influence of individual meteorological factors on local PM2.5 concentration in the Jing-Jin-Ji region. Sci. Rep..

[16] Jiang P., Yang J., Huang C., Liu H. (2018). The contribution of socioeconomic factors to PM2.5 pollution in urban China. Environ. Pollut..

[17] Zhou H., Jiang M., Huang Y., Wang Q. (2021). Directional spatial spillover effects and driving factors of haze pollution in North China Plain. Resour. Conserv. Recycl..

[18] Yang D., Wang X., Xu J., Xu C., Lu D., Ye C., Wang Z., Bai L. (2018). Quantifying the influence of natural and socioeconomic factors and their interactive impact on PM2.5 pollution in China. Environ. Pollut..

[19] Zhang X., Wu Y., Gu B. (2016). Characterization of haze episodes and factors contributing to their formation using a panel model. Chemosphere.

[20] Yang Y., Lan H., Li J. (2020). Spatial Econometric Analysis of the Impact of Socioeconomic Factors on PM2.5 Concentration in China’s Inland Cities: A Case Study from Chengdu Plain Economic Zone. Int. J. Environ. Res. Public Health..

[21] Shi T., Liu M., Hu Y., Li C., Zhang C., Ren B. (2019). Spatiotemporal Pattern of Fine Particulate Matter and Impact of Urban Socioeconomic Factors in China. Int. J. Environ. Res. Public Health..

[22] Zhou S., Lin R. (2019). Spatial-temporal heterogeneity of air pollution: The relationship between built environment and on-road PM2.5 at micro scale. Transport. Res. D-Tr. E.

[23] Liu Q., Wu R., Zhang W., Li W., Wang S. (2020). The varying driving forces of PM2.5 concentrations in Chinese cities: Insights from a geographically and temporally weighted regression model. Environ. Int..

[24] Dong F., Zhang S., Long R., Zhang X., Sun Z. (2019). Determinants of haze pollution: An analysis from the perspective of spatiotemporal heterogeneity. J. Clean. Prod..

[25] Wang S., Liu X., Yang X., Zou B., Wang J. (2018). Spatial variations of PM 2.5 in Chinese cities for the joint impacts of human activities and natural conditions: A global and local regression perspective. J. Clean. Prod..

[26] An Z., Huang R.-J., Zhang R., Tie X., Li G., Cao J., Zhou W., Shi Z., Han Y., Gu Z. (2019). Severe haze in northern China: A synergy of anthropogenic emissions and atmospheric processes. Proc. Natl. Acad. Sci. USA..

[27] Ding Y., Zhang M., Qian X., Li C., Chen S., Wang W. (2019). Using the geographical detector technique to explore the impact of socioeconomic factors on PM2.5 concentrations in China. J. Clean. Prod..

[28] Wu W., Zhang M., Ding Y. (2020). Exploring the effect of economic and environment factors on PM2.5 concentration: A case study of the Beijing-Tianjin-Hebei region. J. Environ. Manag..

[29] Yu X., Geng Y., Dong H., Ulgiati S., Liu Z., Liu Z., Ma Z., Tian X., Sun L. (2016). Sustainability assessment of one industrial region: A combined method of emergy analysis and IPAT (Human Impact Population Affluence Technology). Energy.

[30] Wang S., Zhou C., Wang Z., Feng K., Hubacek K. (2017). The characteristics and drivers of fine particulate matter (PM2.5) distribution in China. J. Clean. Prod..

[31] Zhang L., An J., Liu M., Li Z., Liu Y., Tao L., Liu X., Zhang F., Zheng D., Gao Q. (2020). Spatiotemporal variations and influencing factors of PM2.5 concentrations in Beijing, China. Environ. Pollut..

[32] Zhang H., Zhan Y., Li J., Chao C.-Y., Liu Q., Wang C., Jia S., Ma L., Biswas P. (2021). Using Kriging incorporated with wind direction to investigate ground-level PM2.5 concentration. Sci. Total Environ..

[33] Ehrlich P.R., Holdren J.P. (1971). Impact of population growth. Science.

[34] Chertow M.R. (2000). The IPAT equation and its variants. J. Ind. Ecol..

[35] Xue W., Zhang J., Zhong C., Li X., Wei J. (2021). Spatiotemporal PM2.5 variations and its response to the industrial structure from 2000 to 2018 in the Beijing-Tianjin-Hebei region. J. Clean. Prod..

[36] Jia R., Fan M., Shao S., Yu Y. (2021). Urbanization and haze-governance performance: Evidence from China’s 248 cities. J. Environ. Manag..

[37] Ji X., Yao Y., Long X. (2018). What causes PM2.5 pollution? Cross-economy empirical analysis from socioeconomic perspective. Energy Policy.

[38] Wang Z.-B., Fang C.-L. (2016). Spatial-temporal characteristics and determinants of PM2.5 in the Bohai Rim Urban Agglomeration. Chemosphere.

[39] Zhang N.-N., Ma F., Qin C.-B., Li Y.-F. (2018). Spatiotemporal trends in PM2.5 levels from 2013 to 2017 and regional demarcations for joint prevention and control of atmospheric pollution in China. Chemosphere.

[40] Chen T., Li M., Luo L., Deng S., Zhou R., Chen D. (2020). Simulating the effects of land urbanization on regional meteorology and air quality in Yangtze River Delta, China. Appl. Geogr..

[41] Zhang H., Wang Y., Hu J., Ying Q., Hu X.-M. (2015). Relationships between meteorological parameters and criteria air pollutants in three megacities in China. Environ. Res..

[42] Yang Y., Russell L.M., Lou S., Liao H., Guo J., Liu Y., Singh B., Ghan S.J. (2017). Dust-wind interactions can intensify aerosol pollution over eastern China. Nat. Commun..

[43] Chen Z., Chen D., Zhao C., Kwan M.-p., Cai J., Zhuang Y., Zhao B., Wang X., Chen B., Yang J. (2020). Influence of meteorological conditions on PM2.5 concentrations across China: A review of methodology and mechanism. Environ. Int..

[44] Fan L., Fu S., Wang X., Fu Q., Jia H., Xu H., Qin G., Hu X., Cheng J. (2021). Spatiotemporal variations of ambient air pollutants and meteorological influences over typical urban agglomerations in China during the COVID-19 lockdown. J. Environ. Sci..

[45] Bai H., Gao W., Zhang Y., Wang L. (2021). Assessment of health benefit of PM2.5 reduction during COVID-19 lockdown in China and separating contributions from anthropogenic emissions and meteorology. J. Environ. Sci..

[46] Guo H., Wang Y., Zhang H. (2017). Characterization of criteria air pollutants in Beijing during 2014–2015. Environ. Res..

[47] Wang J., Xu C. (2017). Geodetector: Principle and prospective. Acta Geogr. Sin..

[48] Wang J., Li X., Christakos G., Liao Y., Zhang T., Gu X., Zheng X. (2010). Geographical Detectors-Based Health Risk Assessment and its Application in the Neural Tube Defects Study of the Heshun Region, China. Int. J. Geogr. Inf. Sci..

[49] Bai L., Jiang L., Yang D., Liu Y. (2019). Quantifying the spatial heterogeneity influences of natural and socioeconomic factors and their interactions on air pollution using the geographical detector method: A case study of the Yangtze River Economic Belt, China. J. Clean. Prod..

[50] Fotheringham S., Charlton M., Brunsdon C. (1996). The geography of parameter space: An investigation of spatial non-stationarity. Int. J. Geogr. Inf. Sci..

[51] Huang B., Wu B., Barry M. (2010). Geographically and temporally weighted regression for modeling spatio-temporal variation in house prices. Int. J. Geogr. Inf. Sci..

[52] Mi Y., Sun K., Li L., Lei Y., Wu S., Tang W., Wang Y., Yang J. (2021). Spatiotemporal pattern analysis of PM2.5 and the driving factors in the middle Yellow River urban agglomerations. J. Clean. Prod..

[53] Deng C., Qin C., Li Z., Li K. (2022). Spatiotemporal variations of PM2.5 pollution and its dynamic relationships with meteorological conditions in Beijing-Tianjin-Hebei region. Chemosphere.

[54] Xu M., Qin Z., Zhang S. (2021). Integrated assessment of cleaning air policy in China: A case study for Beijing-Tianjin-Hebei region. J. Clean. Prod..

[55] Xu M., Qin Z., Zhang S., Xie Y. (2021). Health and economic benefits of clean air policies in China: A case study for Beijing-Tianjin-Hebei region. Environ. Pollut..

[56] Wang Y., Liu C., Wang Q., Qin Q., Ren H., Cao J. (2021). Impacts of natural and socioeconomic factors on PM2.5 from 2014 to 2017. J. Environ. Manag..

[57] Chen Z., Xie X., Cai J., Chen D., Gao B., He B., Cheng N., Xu B. (2018). Understanding meteorological influences on PM2.5 concentrations across China: A temporal and spatial perspective. Atmos. Chem. Phys..

[58] Zhang Y., Wang W., Liang L., Wang D., Cui X., Wei W. (2020). Spatial-temporal pattern evolution and driving factors of China’s energy efficiency under low-carbon economy. Sci. Total Environ..

[59] Zhang B., Jiao L., Xu G., Zhao S., Tang X., Zhou Y., Gong C. (2018). Influences of wind and precipitation on different-sized particulate matter concentrations (PM2.5, PM10, PM2.5-10). Meteorol. Atmos. Phys..

[60] Liu Q., Wang S., Zhang W., Li J., Dong G. (2019). The effect of natural and anthropogenic factors on PM2.5: Empirical evidence from Chinese cities with different income levels. Sci. Total Environ..

[61] Wang J., Ogawa S. (2015). Effects of Meteorological Conditions on PM2.5 Concentrations in Nagasaki, Japan. Int. J. Environ. Res. Public Health..

[62] Wu Z., Zhang S. (2019). Study on the spatial-temporal change characteristics and influence factors of fog and haze pollution based on GAM. Neural. Comput. Appl..

[63] Li X., Song H., Zhai S., Lu S., Kong Y., Xia H., Zhao H. (2019). Particulate matter pollution in Chinese cities: Areal-temporal variations and their relationships with meteorological conditions (2015-2017). Environ. Pollut..

[64] Zhang Y. (2019). Dynamic effect analysis of meteorological conditions on air pollution: A case study from Beijing. Sci. Total Environ..

[65] Fu X., Wang X., Hu Q., Li G., Ding X., Zhang Y., He Q., Liu T., Zhang Z., Yu Q. (2016). Changes in visibility with PM2.5 composition and relative humidity at a background site in the Pearl River Delta region. J. Environ. Sci..

[66] Han B., Zhang R., Yang W., Bai Z., Ma Z., Zhang W. (2016). Heavy haze episodes in Beijing during January 2013: Inorganic ion chemistry and source analysis using highly time-resolved measurements from an urban site. Sci. Total Environ..

[67] Zhou B., Shen H., Huang Y., Li W., Chen H., Zhang Y., Su S., Chen Y., Lin N., Zhuo S. (2015). Daily variations of size-segregated ambient particulate matter in Beijing. Environ. Pollut..

[68] Li J., Chen H., Li Z., Wang P., Cribb M., Fan X. (2015). Low-level temperature inversions and their effect on aerosol condensation nuclei concentrations under different large-scale synoptic circulations. Adv. Atmos. Sci..

[69] Liu C.-N., Lin S.-F., Tsai C.-J., Wu Y.-C., Chen C.-F. (2015). Theoretical model for the evaporation loss of PM2.5 during filter sampling. Atmos. Environ..

[70] Lee G., Oh H., Ho C., Park D.R., Kim J., Chang L., Lee J., Choi J., Sung M. (2018). Slow Decreasing Tendency of Fine Particles Compared to Coarse Particles Associated with Recent Hot Summers in Seoul, Korea. Aerosol. Air Qual. Res..

[71] Dong F., Yu B., Pan Y. (2019). Examining the synergistic effect of CO2 emissions on PM2.5 emissions reduction: Evidence from China. J. Clean. Prod..

[72] Chen J., Wang B., Huang S., Song M. (2020). The influence of increased population density in China on air pollution. Sci. Total Environ..

[73] Jiang W., Gao W., Gao X., Ma M., Zhou M., Du K., Ma X. (2021). Spatio-temporal heterogeneity of air pollution and its key influencing factors in the Yellow River Economic Belt of China from 2014 to 2019. J. Environ. Manag..

[74] Zhou C., Chen J., Wang S. (2018). Examining the effects of socioeconomic development on fine particulate matter (PM2.5) in China’s cities using spatial regression and the geographical detector technique. Sci. Total Environ..

[75] Wang X., Tian G., Yang D., Zhang W., Lu D., Liu Z. (2018). Responses of PM2.5 pollution to urbanization in China. Energy Policy.

[76] Zhang X., Gu X., Cheng C., Yang D. (2020). Spatiotemporal heterogeneity of PM2.5 and its relationship with urbanization in North China from 2000 to 2017. Sci. Total Environ..

[77] Luo K., Li G., Fang C., Sun S. (2018). PM2.5 mitigation in China: Socioeconomic determinants of concentrations and differential control policies. J. Environ. Manag..

[78] Guan D., Su X., Zhang Q., Peters G.P., Liu Z., Lei Y., He K. (2014). The socioeconomic drivers of China’s primary PM2.5 emissions. Environ. Res. Lett..

[79] Du Y., Sun T., Peng J., Fang K., Liu Y., Yang Y., Wang Y. (2018). Direct and spillover effects of urbanization on PM2.5 concentrations in China’s top three urban agglomerations. J. Clean. Prod..

[80] Cheng L., Zhang T., Chen L., Li L., Wang S., Hu S., Yuan L., Wang J., Wen M. (2020). Investigating the Impacts of Urbanization on PM2.5 Pollution in the Yangtze River Delta of China: A Spatial Panel Data Approach. Atmosphere.

[81] Xu W., Sun J., Liu Y., Xiao Y., Tian Y., Zhao B., Zhang X. (2019). Spatiotemporal variation and socioeconomic drivers of air pollution in China during 2005-2016. J. Environ. Manag..

[82] Cheng Z., Li L., Liu J. (2020). The impact of foreign direct investment on urban PM2.5 pollution in China. J. Environ. Manag..

[83] Wang T., Peng J., Wu L. (2021). Heterogeneous effects of environmental regulation on air pollution: Evidence from China’s prefecture-level cities. Environ. Sci. Pollut. Res..

[84] Zhao N., Zhang Y., Li B., Hao J., Chen D., Zhou Y., Dong R. (2019). Natural gas and electricity: Two perspective technologies of substituting coal-burning stoves for rural heating and cooking in Hebei Province of China. Energy Sci. Eng..

[85] Guo X., Ren D., Li C. (2020). Study on clean heating based on air pollution and energy consumption. Environ. Sci. Pollut. Res..

[86] Yan D., Kong Y., Jiang P., Huang R., Ye B. (2021). How do socioeconomic factors influence urban PM2.5 pollution in China? Empirical analysis from the perspective of spatiotemporal disequilibrium. Sci. Total Environ..

[87] Wang J., Wang S., Li S. (2019). Examining the spatially varying effects of factors on PM2.5 concentrations in Chinese cities using geographically weighted regression modeling. Environ. Pollut..

[88] Braungardt S., Elsland R., Eichhammer W. (2016). The environmental impact of eco-innovations: The case of EU residential electricity use. Environ. Econ. Policy..

[89] Song M., Fisher R., Kwoh Y. (2019). Technological challenges of green innovation and sustainable resource management with large scale data. Technol. Forecast. Soc..

[90] Gupta H., Barua M.K. (2018). A framework to overcome barriers to green innovation in SMEs using BWM and Fuzzy TOPSIS. Sci. Total Environ..

[91] Ghisetti C., Mancinelli S., Mazzanti M., Zoli M. (2017). Financial barriers and environmental innovations: Evidence from EU manufacturing firms. Clim. Policy.

